# Determinants of disability development in patients with multiple sclerosis

**DOI:** 10.1590/0004-282X-ANP-2020-0338

**Published:** 2021-06-23

**Authors:** Fatma Kara, Mehmet Fatih Göl, Cavit Boz

**Affiliations:** 1 Karadeniz Technical University Faculty of Medicine Department of Neurology Trabzon Turkey Karadeniz Technical University, Faculty of Medicine, Department of Neurology, Trabzon, Turkey.

**Keywords:** Multiple Sclerosis, Epidemiology, Therapy, Esclerose Múltipla, Epidemiologia, Terapia

## Abstract

**Background:**

Multiple sclerosis (MS) is one of the most common chronic neurological diseases affecting the central nervous system in young adults.

**Objective:**

To investigate demographic and clinical factors that are effective in the development of irreversible disability from the onset of MS, and to identify factors that affect the transformation from the relapse-remitting MS (RRMS) phase to the progressive MS (PMS) phase.

**Methods:**

Retrospective study on 741 patients who were diagnosed with RRMS and PMS according to the McDonald criteria, and were enrolled into the Turkish MS database of the Department of Neurology MS Polyclinic, at the Faculty of Medicine, Karadeniz Technical University, in Trabzon, Turkey. Kaplan-Meier analysis was used to evaluate the time taken to reach EDSS 4 and EDSS 6 from the onset of disease, and the time taken between EDSS 4 and EDSS 6.

**Results:**

Age of onset >40 years; having polysymptomatic-type onset, pyramidal or bladder-intestinal system-related first episode; ≥7 episodes in the first 5 years; and <2 years between the first two episodes were found to be effective for MS patients to reach EDSS 4 and EDSS 6. The demographic and clinical parameters that were effective for progression from EDSS 4 to EDSS 6 were: pyramidal or bladder-intestinal system-related first episode; 4‒6 episodes in the first 5 years; >2 years until start of first treatment; and smoking.

**Conclusions:**

Our findings reveal important characteristics of MS patients in our region. However, the associations between these parameters and MS pathophysiology remain to be elucidated.

## RESUMO

**Introdução:**

A esclerose múltipla (EM), uma das doenças neurológicas crônicas mais comuns, afeta o sistema nervoso central em jovens adultos.

**Objetivo:**

Investigar fatores demográficos e clínicos que são efetivos no desenvolvimento de deficiência irreversível, desde o início da EM, e identificar fatores que afetam a transformação da fase de EM recorrente-remitente (EMRR) para a fase de EM secundária progressiva (EMSP).

**Métodos:**

Estudo retrospectivo de 741 pacientes que foram diagnosticados com EMRR e EMSP, de acordo com os critérios de McDonald, e inscritos no banco de dados turco MSBase, do Departamento de Neurologia da MS Polyclinic, da Universidade Técnica de Karadeniz, Turquia. Análise de Kaplan-Meier foi usada para avaliar o tempo para alcançar EDSS 4 e EDSS 6, desde o início da doença e o tempo entre EDSS 4 e EDSS 6.

**Resultados:**

Idade de início>40 anos, início do tipo polissintomático, primeiro ataque relacionado ao sistema piramidal ou bexiga-intestinal, ≥7 recaídas nos primeiros 5 anos e <2 anos entre os dois primeiros ataques foram considerados eficazes em pacientes com EM que atingiram EDSS 4 e EDSS 6. Parâmetros demográficos e clínicos que foram efetivos no progresso de EDSS 4 para EDSS 6: primeiro ataque relacionado ao sistema piramidal ou bexiga-intestinal, 4‒6 recaídas nos primeiros 5 anos, >2 anos até o início do primeiro tratamento e tabagismo.

**Conclusão:**

Estudo revelou características importantes dos pacientes com EM em nossa região. No entanto, as associações entre esses parâmetros e a fisiopatologia da EM ainda precisam ser elucidadas.

## INTRODUCTION

Multiple sclerosis (MS) is one of the most common chronic neurological diseases affecting the central nervous system (CNS) in young adults. Its frequency is higher among females^1^. MS progresses with inflammatory demyelination and subsequent axonal loss, which causes a wide range of unpredictable functional disabilities that are associated with the CNS region(s) affected^2^^,^^3^. It was reported in 2013 that around 2.3 million people suffer from MS on a global scale^4^.

There are three types of MS. The first and the most common type is relapsing-remitting multiple sclerosis (RRMS), which is characterised by relapses of new or increasing neurological symptoms that are followed by periods of partial or complete recovery. The secondary progressive (SPMS) course develops in RRMS patients around 15‒20 years after onset. The third type is primary progressive multiple sclerosis (PPMS), which shows steady progression from the onset for at least 6 months or more without attacks^5^.

Clinical variability during the onset of MS may provide important prognostic clues about the progression of the disease to the progressive stage. Factors associated with a good prognosis in MS include early onset, female sex, initial symptoms consisting of sensory symptoms or optic neuritis, cases in which the initial symptom affects only one CNS region, having less disability at 5 years after onset, better recovery after the first episode, longer duration between first and second episode, low number of episodes during the first five years and a long time until the Expanded Disability Status Scale (EDSS) score exceeds 3 points. On the other hand, the following factors are considered to suggest poor prognosis: male sex, advanced age, onset with motor-cerebellar-spinal cord or bladder-intestinal tract symptoms, incomplete recovery after the first episode, high relapse rate in the first two years, severe disability at 5 years after onset, a short time until EDSS score exceeds 3 points and a short time between the first and second episodes^6^^,^^7^. Because the disease is unpredictable in nature and the factors affecting prognosis vary from region to region^8^^,^^9^^,^^10^, determining the characteristics of disease progression and factors that contribute to disabilities are of high importance for clinical management.

In this study, we aimed to determine the demographic and clinical factors that are effective for development of irreversible disability from the onset of disease, and to identify factors that affect the transformation from the RRMS phase to the secondary progressive MS (SPMS) phase.

## METHODS

### Patients

This was a retrospective study on 741 patients who were diagnosed with RRMS and PMS in accordance with the McDonald criteria^11^. This study analyzed a cohort of Turkish people: demographic and clinical data were collected from the medical records of the Turkish MS Registry at Farabi Hospital, Faculty of Medicine, Karadeniz Technical University, in Trabzon, Turkey. The patients were followed up on a regular basis, with at least one visit every 3-4 months, and the patients were also examined when they came back to the hospital during periods of active complaints. Approval for the study was obtained from Karadeniz Technical University Faculty of Medicine Ethics Council.

#### Inclusion criteria

- Patients diagnosed with RRMS and SPMS in accordance with the McDonald 2010 criteria.
- Signing the consent form and agreeing to participate and continue in the study.

#### Exclusion criteria

- Diagnoses of PPMS and progressive relapsing MS (PRMS) (those with progressive-type onset).
- Diagnoses of any other neurological or psychological disease.
- Not signing the consent form.

### Study design

Forms prepared by the researchers were used to determine the clinical features of the disease and the sociodemographic characteristics of the patients. The following were recorded on the patient preliminary information form: patient's name and sex, disease history, family history, patient's complaints on admission, date of disease onset, number of episodes, date and time of episodes, history of episodes, episode treatments, medications used in MS treatment, treatment duration, other physical diseases and medications used, EDSS score, diagnostic tools and history of drug and substance use. These forms were filled out when the patient first sought care, and the forms were also updated at the times of patient follow-ups.

The following parameters were evaluated for associations with disease progression: age at onset, sex, type of onset (determined as monosymptomatic or polysymptomatic),type of first episode, number of episodes in the first five years after the onset of the disease, length of time between the first and second episodes, time of starting immunomodulator or immunosuppressive treatments that affect disease progression, smoking (and number of cigarettes smoked per day), presence of EDSS 4 (limitation of walking without restriction) and EDSS 6 (walking with one-sided support), and the time taken to reach specific degrees of disability, such as progression from EDSS 4 to EDSS 6.

In this study, the patients were classified according to the first attack types, into five categories: sensory, visual, pyramidal, brainstem-cerebellar and bladder-intestinal. They were classified based on their age at onset of the disease, into the following three categories: <18, 18‒40 or >40 years. They were classified based on the number of relapses in the first five years, into the following three groups: 1‒3, 4‒6 and ≥7 relapses. The patients were divided into three groups according to the time interval between the first two attacks (<2, 2‒5 and >5 years) and they were also divided into two groups according to the starting time of treatment (≤2 years and >2 years).

In MS, an episode (a period of worsening) is defined as a period wherein new symptoms develop or existing symptoms are exacerbated, or a period in which new neurological findings lasting for at least 24 hours (often ending with partial or complete recovery) are observed. Symptoms occurring within one month are considered as part of the same episode. The progressive phase is defined as continuous worsening of symptoms and indications for at least 6 months, with or without the occurrence of episodes, that causes an irreversible increase of at least 1.0 point in EDSS score when it was previously ≤5.5 or 0.5 point when it was >5.5 (increases in EDSS score in the period related to relapses and independent from corticosteroid treatment response were ruled out)^4^^,^^6^.

### Statistical analysis

The data obtained from the study were transferred to electronic media and analyzed using the SPSS 20.0 statistical software package. In addition to descriptive statistical methods (mean, standard deviation, median, frequency, ratio, minimum and maximum), quantitative data were analyzed by means of statistical tests. Chi-square analysis was used to evaluate whether there was any difference between two or more groups, whether there was any correlation between the two variables and the degree of homogeneity between groups. In the chi-square analysis used in our study, the endpoints were defined as reaching EDSS 4, reaching EDSS 6, progression from EDSS 4 to EDSS 6, and reaching the secondary progressive phase of the disease. Kaplan-Meier analysis was also used to evaluate the times taken to reach EDSS 4 and EDSS 6 from the onset of disease, and the length of time between EDSS 4 and EDSS 6. The statistical significance level was determined as p<0.05 in all analyses.

## RESULTS

### Demographic and clinical characteristics of multiple sclerosis patients

Out of the 926 MS patients registered in the Turkish MS database, 741 patients were included in this study and 185 MS patients were excluded. These were excluded because of their progressive onset, missing EDSS scores or irregular follow-up. There were 235 patients with follow-ups shorter than 5 years, 450 MS patients with follow-ups shorter than 10 years and 291 MS patients with follow ups of more than 10 years. The demographic and clinical characteristics of the MS patients are shown in Table 1.

**Table 1:** Demographic and clinical features of multiple sclerosis patients.

Mean±SD or n/%)		RRMS (n=645)	PMS (n=96)	p-value
Sex				
	Male	224 (34.7%)	31 (32.3%)	0.639
	Female	421 (65.3%)	65 (67.7%)	
Educational level				
	Illiterate	12 (2%)	5 (5.7%)	<0.001*
	Primary school	256 (41.6%)	60 (69.0%)	
	High school	161 (26.2%)	14 (16.1%)	
	University	186 (30.2%)	8 (9.2%)	
Marital status				
	Single	162 (26.2%)	6 (68%)	<0.001*
	Married	451 (72.9%)	79 (89.8%)	
	Divorced	6 (1%)	3 (3.4%)	
Age (years)		39.34 (±10.67)	50.04 (±8.64)	<0.001*
Age at onset (years)		30.03 (±10.11)	31.56 (±10.31)	0.168
Duration of disease (years)		7.65 (±7)	8.32 (±6.11)	0.073
Disease onset				
	Monosymptomatic	591 (91.6%)	70 (72.9%)	<0.001*
	Polysymptomatic	54 (8.4%)	26 (27.1%)	
First attack type				
	Visual	160 (24.8%)	13 (13.5%)	<0.001*
	Sensory	147 (22.8%)	4 (4.2%)	
	Pyramidal	176 (27.3%)	56 (58.3%)	
	Brainstem-cerebellar	155 (24%)	21 (21.9%)	
	Bladder-intestinal	7 (1.1%)	2 (2.1%)	
Number of attacks in 5 years		2.79 (±1.52)	5.18 (±1.77)	<0.001*
Time between first two attacks (months)		33 (±45)	25.5 (±38.7)	0.046*
Treatment start time (months)		46.2 (±64.8)	94.4 (±89)	<0.001*
Smoking				
	Yes	172 (90.1%)	19 (9.9%)	0.991
	No	314 (90.5%)	33 (9.5%)	
Number of cigarettes per day				
	1-10	44 (32.6%)	6 (50%)	0.340
	≥10	91 (67.4%)	6 (50%)	
Types of disease-modifying treatment				
First-line therapies				
	Interferon beta-1a	261 (40.5%)	40 (41.7%)	
	Interferon beta-1b	120 (18.6%)	21 (21.9%)	
	Glatiramer acetate	129 (20%)	20 (20.8%)	
Second-line therapies				
	Azathioprine	3 (0.4%)	10 (10.4%)	>0.05
	Fingolimod	34 (5.3%)	-	
	Teriflunomide	23 (3.6%)	-	
	Methotrexate	-	2 (2.1%)	
	Mitoxantrone	-	2 (2.1%)	
	Natalizumab	-	1 (1%)	
	Cyclophosphamide			
Not receiving any treatment		74 (11.5%)	-	

RRMS: relapse-remitting multiple sclerosis.

Among the 741 MS patients who met the inclusion criteria, 65.6% (n=486) were female and 34.4% (n=255) were male. The female/male ratio was 1.9. Among these patients, 87% (n=645) had a diagnosis of RRMS and 13% (n=96) had a diagnosis of PMS (Table 1).

Evaluation of the descriptive statistics on the demographic and clinical characteristics of the patients with RRMS and PMS showed that low education level (p<0.001), being single (p<0.001), higher age (p<0.001), polysymptomatic onset (p<0.001), having an initial episode with pyramidal or bladder-intestinal symptoms (p<0.001), higher number of episodes in first 5 years (p<0.001), short time period between the first two episodes (p=0.046) and longer time period until the start of the first treatment (p<0.001) were associated with worse clinical progress. The results from the chi-square analysis on the demographic and clinical characteristics of MS patients are shown in Table 2.

**Table 2:** Distribution of progression among multiple sclerosis patients.

		Total	EDSS 4	p-value	EDSS 6	p-value	Progressive phase	p-value	EDSS 4-6	p-value
Sex										
	Male	486	143 (29.4%)	0.273	83 (17.1%)	0.554	65 (13.4%)	0.639	77 (53.8%)	0.968
	Female	255	85 (33.3%)		48 (18.8%)		31 (12.2%)		46 (54.1%)	
Age at onset										
	<18	59	21 (35.6%)	0.069	8 (13.6%)	0.398	6 (10.2%)	0.277	7 (33.3%)	0.102
	18-40	560	160 (28.6%)		97 (17.3%)		69 (12.3%)		92 (57.5%)	
	>40	122	47 (38.5%)		26 (21.3%)		21 (17.2%)		24 (51.1%)	
Disease onset										
	Monosymptomatic	661	186 (28.1%)	<0.001*	101 (15.3%)	<0.001*	70 (10.6%)	<0.001*	95 (51.1%)	0.097
	Polysymptomatic	80	42 (52.5%)		30 (37.5%)		26 (32.5%)		28 (66.7%)	
First attack type										
	Visual	173	43 (24.9%)	<0.001*	22 (12.7%)	<0.001*	13 (7.5%)	<0.001*	18 (41.9%)	0.005*
	Brainstem-cerebellar	176	48 (27.3%)		26 (14.8%)		21 (11.9%)		26 (54.2%)	
	Pyramidal	232	112 (48.3%)		74 (31.9%)		56 (24.1%)		70 (62.5%)	
	Sensory	151	22 (14.6%)		6 (4%)		4 (2.6%)		6 (27.3%)	
	Bladder-intestinal	9	3 (33.3%)		3 (33.3%)		2 (22.2%)		3 (100%)	
Number of attacks in 5 years										
	1-3	519	115 (22.2%)	<0.001*	44 (8.5%)	<0.001*	18(%3.5)	<0.001*	39 (33.9%)	<0.001*
	4-6	174	84 (48.3%)		63 (36.2%)		55(%31.6)		60 (71.4%)	
	≥7	48	29 (60.4%)		24 (50%)		23(%47.9)		24 (82.8)	
Time between first two attacks										
	<2 years	428	136 (31.8%)	0.007*	86 (20.1%)	0.029*	69 (16.1)	0.017*	81 (59.6%)	0.108
	2-5 years	197	47 (23.9%)		23 (11.7%)		18 (8.5%)		20 (42.6%)	
	>5 years	107	44 (41.1%)		22 (20.6%)		9 (9.8%)		22 (50%)	
Time of treatment after diagnosis										
	≤2 years	320	66 (20.6%)	<0.001*	29 (9.1%)	<0.001*	19 (5.9%)	<0.001*	27 (40.9%)	0.009*
	>2 years	340	150 (44.1%)		96 (28.2%)		69 (20.3%)		90 (60%)	
Smoking										
	Yes	191	56 (29.3%)	0.902	34 (17.8%)	0.446	19 (9.9%)	0.991	32 (57.1%)	0.294
	No	347	100 (28.8%)		53 (15.3%)		33 (9.5%)		47 (47%)	
Number of cigarettes per day										
	1-10	50	13 (26%)	1.000	8 (16%)	0.723	6 (12%)	0.340	7 (53.8%)	0.904
	≥10	97	24 (24.7%)		12 (12.4%)		6 (6.2%)		11 (45.8%)	

EDSS: Expanded Disability Status Scale.

The results from Kaplan-Meier analysis on demographic and clinical parameters affecting the progression and development of MS patients are shown in Table 3. It was observed that the demographic and clinical parameters that were effective regarding the time taken to reach EDSS 4 and EDSS 6 were similar. These parameters were as follows: age at onset >40 years; having polysymptomatic-type onset, pyramidal or bladder-intestinal system-related first attack; ≥7 relapses in the first 5 years; and <2 years between the first two attacks. The demographic and clinical parameters that were effective regarding the progress from EDSS 4 to EDSS 6 were: pyramidal or bladder-intestinal system-related first attack; >3 relapses in the first 5 years; >2 years until initiation of first treatment; and smoking.

**Table 3:** Demographic and clinical features affecting progression among multiple sclerosis patients.

		Total	Time taken to reach EDSS 4			Time taken to reach EDSS 6			Time taken to go from EDSS 4 to EDSS 6		
			Median	95%Cl	p-value	Median	95%Cl	p-value	Median	95%Cl	p-value
General		741	15.4	12.9-17.9		21.9	18.8-25.0		3.0	2.5-3.5	
Sex											
	Female	486	15.4	12.9-17.9	0.370	20.2	15.8-24.6	0.595	3.0	2.4-3.7	0.713
	Male	255	14.2	8.6-19.9		23.5	15.8-31.1		2.8	1.9-3.7	
Age at onset											
	<18	59	17.8	8.2-27.5	<0.001*	25.9		0.003*	5.6	0-11.9	0.110
	18-40	560	16.3	12.9-19.7		20.2	16.3-24.1		2.8	2.0-3.5	
	>40	122	10.3	8.7-11.8		15.6	10.5-20.6		3.3	2.6-3.9	
Disease onset											
	Monosymptomatic	661	15.8	13.3-18.4	0.002*	23.5	19.0-27.9	0.001*	3.0	2.4-3.7	0.285
	Polysymptomatic	80	9.1	7.0-11.2		13.0	10.9-15.1		2.9	1.7-4.0	
First attack type											
	Visual	173	19.7	17.8-21.6	<0.001*	24.3	21.2-27.4	<0.001*	3.0	1.5-4.6	0.013*
	Brainstem-cerebellar	176	13.5	7.9-19.2		23.1	13.4-32.8		2.2	1.3-3.1	
	Pyramidal	232	10.2	8.3-12.0		16.4	13-19.8		3.4	2.4-4.4	
	Sensory	151	42.2								
	Bladder-intestinal	9	8.2	0-18.5		10.3	2.7-17.8		2.0	1.8-2.2	
Number of attacks in 5 years											
	1-3	519	19.7	16.6-22.9	<0.001*	28.7	23.9-33.5	<0.001*	5.3	3.2-7.3	0.001*
	4-6	174	10.8	8.2-13.4		15.9	12.4-19.5		2.8	2.5-3.1	
	≥7	48	5.9	1.2-10.7		11.4	9.1-13.6		3.2	1.8-2.5	
Time between first two attacks											
	<2 years	428	11.3	9.3-13.2	<0.001*	16.5	12.9-20.1	<0.001*	2.9	2.3-3.5	0.453
	2-5 years	197	17.1	11.9-22.3		20.2	18.4-22.0		3.0	1.0-5.3	
	>5 years	107	20.7	16.8-24.7		28.7	25.4-32.0		3.5	1.0-6.1	
Time of treatment after diagnosis											
	≤2 years	320	14.2	6.2-22.2	0.210			0.753	5.0	2.9-7.0	0.015*
	>2 years	340	15.4	12.7-18.1		21.1	17.7-24.5		2.7	2.2-3.2	
Smoking											
	Yes	191	14.2	7.4-21.0	0.846	21.1	13.5-28.7	0.243	2.6	1.8-3.3	0.018*
	No	347	15.5	12.8-18.2		20.1	15.2-28.5		4.2	2.3-6.1	
Number of cigarettes per day											
	1-10	50	19.0	3.1-34.9	0.583			0.200	2.7	1.0-4.8	0.470
	≥10	97	15.8	8.4-23.3		21.1			2.8	0-5.6	

EDSS: Expanded Disability Status Scale; 95%CI: 95% confidence interval.

## DISCUSSION

MS is the most common of the diseases that develop due to inflammatory demyelinating events in the CNS. It is a chronic disease that progresses with neuroinflammation and neurodegeneration in the CNS and is considered to be of autoimmune origin. It involves the cortex and deep gray matter, but white matter is usually affected to a higher degree. Demyelination and axonal degeneration associated with MS lesions cause different degrees of disability development in patients.

In the current study, it was found that sex was not an effective factor for disability development. In other studies, the general impression was that the disease had worse prognosis in male patients than in female patients^12^^,^^13^^,^^14^^,^^15^. However, in yet other published studies, male sex was not found to be a factor associated with poor prognosis, as in our study^16^^,^^17^^,^^18^. In our study, the reason why the male patient group was not associated with a poor prognosis was thought to be the similar prevalence of the disease in males and females, and the exclusion of PPMS and PRMS patients with a progressive-type onset, which may have obscured the results.

Compared with other types of episodes, having an initial episode with bladder-intestinal system symptoms was found to be associated with shorter time to reach EDSS 4 and 6, and faster progression from EDSS 4 to EDSS 6. Furthermore, we observed that the type of first episode had a significant effect on disability development. In the literature, four different studies showed results similar to our study, concluding that patients with a first episode related to the bladder-intestinal system were at increased risk of disability development, whereas only one study reported that sphincter symptoms had no independent effect on disability development^19^^,^^20^. However, different results have been obtained in other studies, and some have associated various other symptoms with MS prognosis (for better or for worse)^17^^,^^21^. Although these studies show that there are contradictory results in the literature, our results were similar to those of the majority of studies, and we found that having a first episode related to the bladder-intestinal system was an effective factor in the development of irreversible disability.

In our study, MS patients were divided into three age groups according to disease onset (<18, 18‒40, and over 40 years of age). From evaluating these groups, we concluded that being over 40 years old at diagnosis was a factor associated with increased disability development. Similar to our study, other studies have also found that late onset of disease was associated with poor prognosis^12^^,^^17^^,^^22^^,^^23^^,^^24^. There are a few studies that reported different findings. For instance, in a study by Trojano et al. published in 1995, it was found that late onset was associated with good prognosis; however, the limit for advanced age was identified as 25 years in that study^25^. Similarly, in another published study, the age at onset of the disease was not found to be associated with poor prognosis^18^. Consistent with many other studies in the literature, we determined that late onset of disease was an indicator of worse clinical progress. This may be attributed to the age-related deterioration of repair mechanisms.

Comparison of onset type showed that MS patients with polysymptomatic-type onset reached EDSS 4 and EDSS 6 significantly faster than patients with monosymptomatic-type onset. In the literature, two different studies reported results similar to ours^22^^,^^26^. On the other hand, polysymptomatic-type onset was found to have no effect on the prognosis of MS in several studies^27^. Our findings can be explained by the fact that the involvement of multiple neurological systems at the same time may be a clinical indicator of the involvement of more than one region in the CNS. Therefore, a relationship between the number of lesions and disability development may exist.

We found that patients with ≥7 episodes in the first five years reached EDSS 4 and EDSS 6 in shorter times, and that their progress from EDSS 4 to EDSS 6 was faster than was seen among other patients. Similarly, in other studies, it was concluded that the number of episodes was an effective factor for disability development^1^^,^^12^^,^^16^^,^^22^^,^^27^. In the study by Tremlett et al., it was found that the number of episodes in the first five years had a significant effect on disease progression, but that the number of episodes over the longer term was less important^28^. Consistent with the literature, we found that a high number of episodes in the first five years was associated with disability development.

It was found that patients with <2 years between the first two episodes reached EDSS 4 and EDSS 6 faster, and there was a statistically significant difference, compared with the other two groups. Consistent with our study, other studies have also shown that a short time interval between the first two episodes is indicative of worse disease progression^16^^,^^17^^,^^27^^,^^29^^,^^30^. Almost all studies in the literature suggest results similar to ours.

There was no statistically significant difference in the time taken to reach EDSS 4 and EDSS 6 between the two groups, according to the time when treatment was started, but it was observed that starting the treatment after two years shortened the progression from EDSS 4 to EDSS 6, and this difference was significant. There are studies in the literature showing that starting treatment early was not related to progression of the disease^29^^,^^31^^,^^32^. Similarly, in another study, receiving treatment at any time of the disease was not associated with poor prognosis^18^ In three different studies, it was shown that disease progression measured by the time taken to reach EDSS milestones was slower with IFN β treatment^30^^,^^33^^,^^34^^,^^35^. In another study, it was found that treatments that were effective against the course of the disease that were started during the clinical isolated syndrome stage prevented disability development^36^. These results suggest that treatments that are effective against the course of the disease may have partially positive effects on disability development. However, in our patient group, there were 235 patients with follow-ups shorter than 5 years, 450 MS patients with follow-ups shorter than 10 years and 291 MS patients with follow-ups of more than 10 years. The fact that the time when treatment started was not directly related to disability development can be explained by the different treatment options used by the patients, and by insufficient follow-up duration for many of our patients, which would limit the evaluation of long-term outcomes from treatments.

In our study, smoking was found to have no effect on reaching the EDSS 4 and EDSS 6 milestones, but it was found that it shortened the progression from EDSS 4 to 6, and this difference was statistically significant. Many studies investigating the relationship between smoking and MS progression have suggest that smoking causes disease progression^37^^,^^38^^,^^39^^,^^40^. The reason why the results from our study were partially consistent with the literature was thought to be related to the lack of smoking data in most patient records included in this study and the possibility that patients provided incomplete or incorrect information about smoking.

In conclusion, in this study, some factors that cause worse disease progression in MS patients were identified. To summarize, these factors were: age at onset (being older than 40 years); polysymptomatic-type onset; having a first-episode type related to the bladder-intestinal system; ≥7 episodes in the first 5 years; <2 years between the first two episodes; time until the start of treatment greater 2 years; and smoking.
